# Delaying the start of iron until 28 days after antimalarial treatment is associated with lower incidence of subsequent illness in children with malaria and iron deficiency

**DOI:** 10.1371/journal.pone.0183977

**Published:** 2017-08-30

**Authors:** Ericka G. Jaramillo, Ezekiel Mupere, Robert O. Opoka, James S. Hodges, Troy C. Lund, Michael K. Georgieff, Chandy C. John, Sarah E. Cusick

**Affiliations:** 1 Icahn School of Medicine at Mount Sinai, New York, New York, United States of America; 2 Department of Paediatrics and Child Health, Makerere University, Kampala, Uganda; 3 Division of Biostatistics, University of Minnesota School of Public Health, Minneapolis, Minnesota, United States of America; 4 Department of Pediatrics, University School of Medicine, Minneapolis, Minnesota, United States of America; 5 Department of Pediatrics, Indiana University, Indianapolis, Indiana, United States of America; University of Ottawa, CANADA

## Abstract

We evaluated the incidence of all-cause and malaria-specific clinic visits during follow-up of a recent trial of iron therapy. In the main trial, Ugandan children 6–59 months with smear-confirmed malaria and iron deficiency [zinc protoporphyrin (ZPP > = 80 μmol/mol heme)] were treated for malaria and randomized to start a 27-day course of oral iron concurrently with (immediate group) or 28 days after (delayed group) antimalarial treatment. All children were followed for the same 56-day period starting at the time of antimalarial treatment (Day 0) and underwent passive and active surveillance for malaria and other morbidity for the entire follow-up period. All ill children were examined and treated by the study physician. In this secondary analysis of morbidity data from the main trial, we report that although the incidence of malaria-specific visits did not differ between the groups, children in the immediate group had a higher incidence rate ratio of all-cause sick-child visits to the clinic during the follow-up period (Incidence Rate Ratio (IRR) immediate/delayed = 1.76; 95%CI: 1.05–3.03, p = 0.033). Although these findings need to be tested in a larger trial powered for malaria-specific morbidity, these preliminary results suggest that delaying iron by 28 days in children with coexisting malaria and iron deficiency is associated with a reduced risk of subsequent all-cause illness.

## Introduction

Malaria is endemic in many regions where iron deficiency is prevalent. The conditions frequently co-occur in the same child. The World Health Organization standard-of-care regimen for treating children with malaria and iron deficiency is to give antimalarial treatment and iron therapy concurrently [1,2]. However, studies following this regimen have reported unresolved anemia and increased risk of infection [3–5].

Increased concentrations of the hepatic protein hepcidin that accompany a malaria episode may explain the findings of unresolved anemia. Hepcidin rises with malaria infection, impairing intestinal iron absorption and release of iron from reticuloendothelial stores [6]. Because elevated hepcidin normalizes approximately four weeks after antimalarial treatment [7], oral iron given before this time may not be well absorbed or utilized. In a recent randomized trial, we demonstrated with iron stable isotopes that starting iron 28 days after, rather than concurrently with, antimalarial treatment in children with malaria and iron deficiency was associated with a two-fold increase of iron incorporation into hemoglobin [8]. However, iron status was equivalent between the groups at the end of the 56-day follow up period, thus demonstrating no clear, short-term benefit or harm of delaying iron on iron status.

Whether a 28-day delay in the start of iron therapy might affect subsequent morbidity is unknown. Iron that is better absorbed would result in less iron trapped in the intestine and available to pathogenic bacteria. Alternatively, better iron absorption may result in more iron-rich blood cells, shown *in vitro* to be preferred by *P*. *falciparum* [9]. Either scenario could increase infection risk.

To determine whether delaying iron until 28 days after antimalarial treatment in children with coexisting iron deficiency and malaria is associated with a difference in the risk of subsequent illness compared to the standard of care concurrent iron therapy, we analyzed morbidity data from our previous trial to investigate the frequency and incidence of physician-diagnosed episodes of illness over the 56-day follow-up period.

## Subjects and methods

### Study population

**Fig 1:**
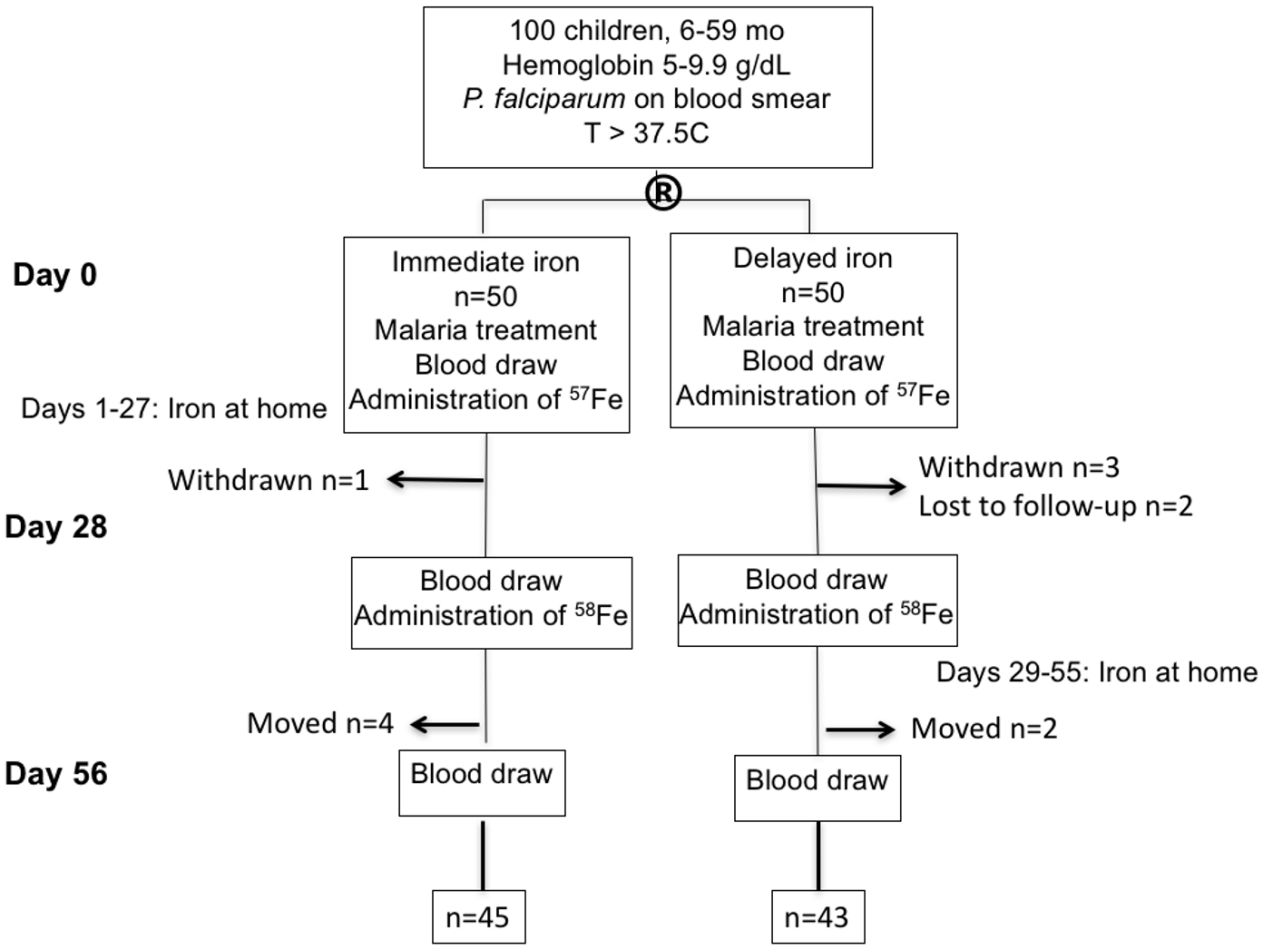
Consort diagram from original study [8].

As described previously [8], 100 children 6–59 months old with malaria (positive Giemsa smear and T>37.5°C) and hemoglobin 5.0–9.9 g/dL were enrolled in a randomized trial of iron therapy at Mulago Hospital in Kampala, Uganda (Fig 1). Children were treated for malaria with parenteral artesunate and also given a 3-day course of oral artemether-lumefantrine. If children had a zinc protoporphyin (ZPP) concentration > = 80 μmol/mol heme, they were randomized either to begin a 27-day iron therapy regimen (2 mg/kg/day as liquid oral ferrous sulfate) concurrently with antimalarial treatment on Day 0 (immediate group, n = 50) or 28 days later on Day 28 (delayed group, n = 50). Children were given an insecticide-treated bednet and were followed for 56 days (Day 0 –Day 56).

During the 56-day follow-up period, all children were under active and passive surveillance for illness. In addition to scheduled clinic visit on Days 28 and 56, home visits were made to each enrolled child’s home by study staff on Days 14 and 42. Any ill child was brought to the study clinic for assessment and treatment. Additionally, all caregivers were instructed to contact the study office in the event of illness that occurred between study visits. Mobile phone airtime was reimbursed for these calls, and transportation to and from the study clinic was provided directly or reimbursed. All medical examination and treatment was provided free of charge. All ill children, whether identified during a home visit or self-referred, were given a physical examination by a study medical officer in the Paediatric Acute Care Unit at Mulago Hospital.

Diagnoses for common illnesses were made according to Mulago Hospital guidelines, including: 1) Uncomplicated malaria: positive Giemsa smear or Rapid Diagnostic Test (RDT), with fever or history of fever, in absence of any of the World Health Organization’s Clinical Signs of Severe Malaria [10]; 2) Severe malaria: positive Giemsa smear or RDT, fever or history of fever, concurrent with one or more clinical signs of severe malaria, including severe anemia, prostration, cerebral malaria, repeated seizures or symptoms like persistent vomiting, high temperature (>39.5°C), or tea-colored urine; 3) Upper respiratory tract infection (URTI): runny nose, cough with or without fever with normal examination chest findings; 4) Lower respiratory tract infection (LRTI): cough, history of fever or fever and examination findings of either respiratory rate that was high for age or crepitations; 5) Otitis media: ear infection; 6) Conjunctivitis: red eyes and discharge; 7) Gastroenteritis: diarrhea (more than 3 loose motions/day) as main symptom, with or without fever or vomiting; 8) Unspecified fevers: fever, negative malaria test, normal complete blood counts with no focus of investigations; 8) Chicken pox: vesicles characteristic of chicken pox (varicella).

For any visit to the study clinic, the findings from the child’s clinical exam, the primary diagnosis and any additional diagnoses were recorded on a sick child visit form and entered into the study’s database.

### Ethical considerations

Caregivers of all enrolled children provided written informed consent. The study was approved by the Institutional Review Board of the University of Minnesota, the School of Medicine Research Ethics Committee at Makerere University, the Uganda National Council of Science and Technology, and the National Drug Authority of Uganda.

### Statistical methods

The original trial was powered to compare the primary outcome of percentage iron incorporation into red blood cells between the immediate vs. delayed groups [8]. This secondary analysis compared the immediate and delayed groups according to the 56-day period prevalence and incidence rate of all-cause morbidity and malaria-specific morbidity, as recorded by sick-child visits to the study clinic. A visit was considered a “malaria visit” if a diagnosis of uncomplicated or severe malaria was made as primary or other diagnosis. For both the all-cause and malaria-specific outcomes, period prevalence was calculated in each group by dividing the number of children with at least one sick visit to the study clinic in the follow-up period by the number of children randomized to the group. We compared the period prevalence between groups using Pearson’s chi-square test. Incidence rates for all-cause and malaria-specific visits were calculated in each group by dividing the total number of sick visits in each group by the number of person-weeks. We compared groups according to incidence rates using Poisson regression with the log link and over-dispersion, which estimated incidence rate ratios. Secondary analyses adjusted these ratios for age, sex, baseline malaria parasite density, baseline hemoglobin, and baseline height-for-age z-score, with each secondary analysis considering one adjuster. Baseline characteristics were compared between the treatment groups using t-tests (age, z-scores) or Wilcoxon rank sum test (malaria parasite density) for continuous outcomes and chi-square for categorical outcomes (sex). Analyses used SPSS version 22.0 (IBM Corp., Armonk, NY), STATA version 12.1 (College Station, TX), and JMP version 12.0 Pro (SAS Institute Inc., Cary, NC).

## Results

The groups did not differ with regard to age, sex, hemoglobin concentration, anthropometry, or malaria parasite density at baseline (Table 1). During the 56-day follow-up period, forty-one children had at least one sick-child visit (Table 2). During the 56-day follow-up period, 28 children (15 immediate, 13 delayed) had one sick visit, 12 children (9 immediate, 3 delayed) had two sick visits, and one child in the immediate group had three sick visits. The mean (range) time to first event was 24.1 (5–54) days in the immediate group and 24.0 days (8–56) days in the delayed group.

**Table 1:** Baseline characteristics of Ugandan children with malaria and anemia by study group^1^.

	Immediate	Delayed	p-value
n	50	50	
Age, years ±SD^2^	2.2 ± 1.2	2.2 ± 1.1	0.79^3^
Sex, male, n (%)	30 (60%)	27 (54%)	0.62^4^
Hemoglobin, g/dL^2^	8.0 (1.5)	8.0 (1.5)	0.82
Height-for-age z-score, ±SD ^2^^,^^5^	-1.4 ±1.1	-1.3 ± 1.0	0.57
HAZ < -2, n (%)	11 (22.9)	10 (21.7)	0.89
Weight-for-height z-score, ±SD^2^^,^^5^	-0.85 ± 1.2	-0.78 ±1.1	0.76
WHZ < -2, n (%)	8 (16.7)	5 (10.8)	0.42
Weight-for-age z-score, ±SD^2^^,^ ^6^	-1.5 ± 1.1	-0.78 ± 1.1	0.59
WAZ < -2, n (%)	19 (38.0)	17 (34.7)	0.73
Malaria parasite density, parasites/μL, [IQR]^7^	46,700 (4600; 111,000)	31,300 (1240; 94,100)	0.40^8^

^1^First published in [8];

^2^Values are means ± SDs

^3^T-test comparing immediate vs. delayed groups (all means)

^4^Chi-square p (all proportions);

^5^Immediate, *n* = 48; Delayed, *n* = 46;

^6^Delayed, *n* = 49;

^7^Values are medians [IQRs]; Immediate, *n* = 46; Delayed, *n* = 43;

^8^Wilcoxon rank sum comparing immediate vs. delayed groups.

HAZ: Height-for-age Z-score; WAZ: Weight-for-age Z-score; WHZ: Weight-for-height Z-score

**Table 2:** Incidence of all-cause and malaria-specific illness^1^.

	Immediate	Delayed	p^2^
**All-cause illness visit**			
Period prevalence^3^	25/50	16/50	0.07
Incidence rate^4^	36/378	19/351	0.03
**Malaria visit**			
Period prevalence	12/50	8/50	0.32
Incidence rate	13/378	9/351	0.49

^1^As assessed by sick-child visits to hospital clinic in the 56-day follow-up period of a recent iron therapy trial that compared the effect on iron status outcomes of iron started concurrently with vs. 28 days after antimalarial treatment in 100 Ugandan children 6–59 months old with malaria and anemia;

^2^P-value from chi-square for period prevalence and Poisson regression for incidence rate;

^3^Period prevalence = children with at least one sick visit in the follow-up period/children enrolled at beginning of study;

^4^Incidence rate = total number of sick visits in follow-up period/person weeks.

The period prevalence, or number of children with at least one illness in the follow-up period divided by the number of children enrolled, did not differ significantly between the groups (p = 0.07, Table 2). However, the incidence rate of all-cause sick-child visits, which accounted for multiple visits per child, was significantly greater among children in the immediate iron group compared to the delayed iron group [Incidence Rate Ratio (IRR) immediate/delayed = 1.76; 95% CI: 1.05–3.03, p = 0.033, Table 2]. For visits in which a malaria diagnosis (uncomplicated or severe) was made, the group comparisons for period prevalence and incidence rate did not reach statistical significance [IRR immediate/delayed = 1.34; 95% CI: 0.59–3.19, p = 0.49]. Adjusting for age, sex, malaria parasite density, hemoglobin, or height-for-age z-score did not significantly change the estimated IRR for either sick child visits or malaria-specific visits (S1 and S2 Tables).

The most frequent diagnoses were upper respiratory tract infection (n = 23), malaria (uncomplicated or severe, n = 22), and gastroenteritis (n = 14, Table 3). The number of children with the most frequently diagnosed illnesses did not differ between groups (immediate vs. delayed; URTI: 12 vs 9, p = 0.46; malaria: 12 vs. 8, p = 0.32; gastroenteritis: 9 vs. 5, p = 0.25). The number of children admitted to the hospital because of their illness also did not differ between groups (8 immediate vs. 6 delayed; p = 0.56). The most common cause of hospitalization was severe malaria (8 children total: 5 immediate vs. 3 delayed, p = 0.72). One child in the immediate group was hospitalized twice for severe malaria.

**Table 3:** Diagnoses of Ugandan children in immediate or delayed iron study^1^.

	Primary Diagnosis	Secondary Diagnosis	Other diagnosis
URTI	16	7	
Uncomplicated malaria	6	4	3
Severe malaria	9		
Gastroenteritis	10	4	
LRTI	9	3	
Skin rash	1	1	1
Chicken pox	1		
Unspecified fever	1	3	
Otitis Media	1	1	
Conjunctivitis		1	
Other	1	5	1

^1^Numbers represent number of times the indicated diagnosis was made as primary, secondary, or other diagnosis for entire study sample and for all visits over the course of the 56-day follow-up period after 100 Ugandan children were treated for malaria and then given a 27-day supply of iron that started immediately or after 28 days.

## Discussion

We did a secondary analysis of morbidity outcomes in a recent randomized trial of iron treatment in which children began a 27-day course of an oral iron supplement concurrently with or 28 days after antimalarial treatment. Our primary finding was that children in the delayed-iron group had lower incidence of all-cause sick-child visits to the study clinic in the 56-day follow-up period compared to children in the immediate-iron group, despite ending the 56-day period with equivalent iron status [8].

Three recent Cochrane reviews [11–13] found no increased risk of malaria with iron supplementation if malaria prevention and management strategies are in place. We also did not find a significant difference between the groups in malaria-specific morbidity, although our study was not powered for this outcome. Similar to a recent study of iron-fortified micronutrient sprinkles in Pakistan, we did find iron to be linked with an increased risk of illnesses other than malaria, including respiratory illness [14].

It is unclear why the incidence of all-cause sick-child visits was greater in our study's immediate group. Perhaps lower absorption of iron at the time of supplementation, which we observed in the immediate iron group [8], translated into more unabsorbed iron in the gut. Unabsorbed iron may shift the gut microbiome in favor of enteropathogenic bacteria, rather than beneficial barrier bifidobacteria [15]. Such a pathogenic shift with iron was observed in Kenyan children receiving iron-fortified food products [15–16] and was associated with more frequent diarrheal illness. Associations between pathogenic shifts in the gut microbiome and exacerbation of respiratory infections have also been described [17–18].

Limitations to this secondary analysis include its small sample size and resulting insufficient power for malaria outcomes. Further, it is important to note that nine children in the immediate group as compared to three children in the delayed group had two episodes of illness. Thus, the difference in incidence rates of all-cause illness between the groups was driven largely by these six children. Although these data are preliminary, they suggest that delaying iron by 28 days after antimalarial treatment is associated with a reduction in all-cause morbidity while not harming iron status. Longer-term studies powered for iron status, morbidity, and neurocognitive developmental outcomes are needed to verify these findings and to determine the safest and most effective management options for children with malaria and iron deficiency.

## Supporting information

S1 Table Adjusted treatment comparisons and adjuster effects for sick-child visits. ^1^IRR for all sick-child visits adjusted for age, sex, malaria parasite density, hemoglobin, or height-for-age z-score; ^2^Standard deviation of the adjuster. HAZ = Height-for-age Z-score, IRR Incidence Rate Ratio.
(DOCX)

S2 Table Adjusted treatment comparisons and adjuster effects for malaria-specific visits. ^1^IRR for malaria-specific visits adjusted for age, sex, malaria parasite density, hemoglobin, or height-for-age z-score; ^2^Standard deviation of the adjuster. HAZ = Height-for-age Z-score, IRR Incidence Rate Ratio.
(DOCX)

S1 File Dataset. (XLSX)

S2 File Original study protocol. (DOCX)
