# Supplementary material for: Delaying the start of iron until 28 days after antimalarial treatment is associated with lower incidence of subsequent illness in children with malaria and iron deficiency
Source: PLoS One. 2017 Aug 30;12(8):e0183977. doi: 10.1371/journal.pone.0183977 (PMC5576757; doi:10.1371/journal.pone.0183977)
Supplement: S1 Table — 1IRR for all sick-child visits adjusted for age, sex, malaria parasite density, hemoglobin, or height-for-age z-score; 2Standard deviation of the adjuster. HAZ = Height-for-age Z-score, IRR Incidence Rate Ratio. (DOCX) [file pone.0183977.s001.docx]

| **Supplemental Table 1:** **Adjusted treatment comparisons and adjuster effects**  **for sick-child visits^1^** | | | | | | | |
| --- | --- | --- | --- | --- | --- | --- | --- |
|  | Treatment comparison | | | Adjuster effect | | | |
| Adjuster | Est IRR | CI | P-value | IRR for | Est | CI | P-value |
| Age | 1.76 | 1.05-3.03 | 0.031 | 1 yr | 0.86 | 0.69-1.07 | 0.17 |
| Sex | 1.72 | 1.03-2.97 | 0.039 | M vs. F | 1.31 | 0.78-2.24 | 0.31 |
| Log malaria parasite density | 1.81 | 1.07-3.15 | 0.028 | 1.09^2^ | 0.92 | 0.72-1.18 | 0.50 |
| Hemoglobin | 1.75 | 1.04-3.04 | 0.034 | 1.53^2^ | 1.08 | 0.84-1.39 | 0.56 |
| HAZ | 1.77 | 1.03-3.14 | 0.038 | 1.04^2^ | 0.93 | 0.71-1.20 | 0.56 |

^1^IRR for all sick-child visits adjusted for age, sex, malaria parasite density, hemoglobin, or height-for-age z-score; ^2^Standard deviation of the adjuster

HAZ = Height-for-age Z-score, IRR Incidence Rate Ratio
