# Supplementary material for: Delaying the start of iron until 28 days after antimalarial treatment is associated with lower incidence of subsequent illness in children with malaria and iron deficiency
Source: PLoS One. 2017 Aug 30;12(8):e0183977. doi: 10.1371/journal.pone.0183977 (PMC5576757; doi:10.1371/journal.pone.0183977)
Supplement: S2 Table — 1IRR for malaria-specific visits adjusted for age, sex, malaria parasite density, hemoglobin, or height-for-age z-score; 2Standard deviation of the adjuster. HAZ = Height-for-age Z-score, IRR Incidence Rate Ratio. (DOCX) [file pone.0183977.s002.docx]

| **Supplemental Table 2: Adjusted treatment comparisons and adjuster effects**  **for malaria-specific visits^1^** | | | | | | | |
| --- | --- | --- | --- | --- | --- | --- | --- |
|  | Treatment comparison | | | Adjuster effect | | | |
| Adjuster | Est IRR | CI | P-value | IRR for | Est | CI | P-value |
| Age | 1.34 | 0.59-3.21 | 0.49 | 1 yr | 0.91 | 0.62-1.28 | 0.58 |
| Sex | 1.39 | 0.61-3.27 | 0.43 | M vs. F | 0.63 | 0.27-1.43 | 0.27 |
| Log malaria parasite density | 1.32 | 0.58-3.12 | 0.51 | 1.09^2^ | 1.10 | 0.73-1.72 | 0.65 |
| Hemoglobin | 1.34 | 0.58-3.21 | 0.49 | 1.53^2^ | 0.96 | 0.63-1.45 | 0.84 |
| HAZ | 1.30 | 0.57-3.09 | 0.54 | 1.04^2^ | 0.86 | 0.55-1.29 | 0.47 |

^1^IRR for malaria-specific visits adjusted for age, sex, malaria parasite density, hemoglobin, or height-for-age z-score; ^2^Standard deviation of the adjuster

HAZ = Height-for-age Z-score, IRR Incidence Rate Ratio
