# Supplementary material for: Delaying the start of iron until 28 days after antimalarial treatment is associated with lower incidence of subsequent illness in children with malaria and iron deficiency
Source: PLoS One. 2017 Aug 30;12(8):e0183977. doi: 10.1371/journal.pone.0183977 (PMC5576757; doi:10.1371/journal.pone.0183977)
Supplement: S2 File — (DOCX) [file pone.0183977.s004.docx]

**Acute vs. Delayed Iron: Effect on Red Cell Iron Incorporation in Severe Malaria**

R03 study protocol

Co-Investigator/Major International Collaborator:

Ezekiel Mupere, MBChB, MMED, MS, PhD

Principal Investigator: Sarah Cusick, Ph.D.

Version 1.2

June 5, 2013

**Key Roles**

**Co-Investigator/Major International Collaborator:**

Ezekiel Mupere, MBChB, MMED, MS, PhD

Senior Lecturer

Department of Paediatrics

Makerere University

College of Health Sciences

School of Medicine

P.O. Box 7072

Kampala, Uganda

Phone: 256 41 531875

Fax: 256 41 532591

Email: [mupez@yahoo.com](mailto:mupez@yahoo.com)

**Principal Investigator**:

Sarah Cusick, PhD

Assistant Professor,

Division of Global Pediatrics

University of Minnesota Medical School

717 Delaware Street, SE

Room 365

Minneapolis, MN 55455

Phone: 612-625-8549

Fax: 612-624-8927

E-mail: scusick@umn.edu

**Other Significant Contributors:**

Chandy C John, MD, MS

Professor of Pediatrics and Medicine

Director, Division of Global Pediatrics

University of Minnesota Medical School

420 Delaware Street, SE

MMC #296, 850-Mayo

Minneapolis, MN 55455

Phone: 612-624-1966

Fax: 612-624-8927

E-mail: ccj@umn.edu

Michael Georgieff, MD

Professor of Pediatrics

Director, Center for Neurobehavioral Development

University of Minnesota Medical School

MMC 39, D-136 Mayo
420 Delaware St. SE
Minneapolis, MN 55455
Phone: 612-626-2971
Fax: 612-624-8176
E-mail: [georg001@umn.edu](mailto:georg001@umn.edu)

Robert OpikaOpoka, MBChB, MMED, MPH

Senior Lecturer

Department of Paediatrics

Mulago Hospital/Makerere University Medical School

Box 7072

Kampala, Uganda

Phone: 256-772-996164

E-mail: [opokabob@yahoo.com](mailto:opokabob@yahoo.com)

**Consultant :**

Steven Abrams, MD

Professor of Pediatrics

BaylorCollege of Medicine

One BaylorPlaza

Houston, TX 77030

Phone : 713-798-4951

Email : sabrams@bcm.edu

**Abstract**

Approximately 1 million children < 5 y living in sub-Saharan Africa die from severe anemia annually. This severe anemia frequently results from coexisting iron deficiency and malaria infection, but the standard of care, concurrent iron therapy and antimalarial treatment, has proven ineffective at curing the profound anemia and has promoted proliferation of the parasite in some studies. The pro-inflammatory immune response mounted against malaria down-regulates iron absorption in the gut, making provision of oral iron supplements during malarial infection of questionable utility. The present study uses iron stable isotopes and a randomized design to test whether starting 4 weeks of iron therapy immediately after antimalarial treatment or 4 weeks later is associated with greater iron incorporation into red blood cells at the time of initial administration of iron therapy and improved long-term hematological recovery. One hundred Ugandan children 6-59 mos with anemia (hemoglobin 5-9.9 g/dL) and with clinical signs of malaria who present to the Pediatric Acute Care Ward of Mulago Hospital in Kampala, Uganda, will be randomized to start iron immediately after antimalarial treatment on Day 0 (immediate group) or 4 weeks later on Day 28 (delayed group). Children will be assessed at the hospital on Day 0, Day 28 and Day 56 and will receive bi-weekly home visits for the 56-day study duration. The specific aims and corresponding hypotheses of the proposed study are:

**Aim 1: Identify the sequencing of antimalarial treatment and iron therapy that results in the greatest red cell iron incorporation at the time of initial iron supplement administration.** The working hypothesis is that red cell iron incorporation will be greater at the time of initial supplement administration in children starting iron 4 weeks after antimalarial treatment (delayed group) compared to children starting iron concurrently with antimalarial treatment (immediate group), due to more complete parasite clearance and resolution of inflammation, permitting better iron uptake, distribution, and utilization.

**Aim 2: Determine whether long-term hematological recovery is impacted by immediate vs. delayed iron.** The working hypothesis is that delayed iron treatment will be associated with greater hemoglobin and improved iron status at Day 56 compared to immediate treatment due to more complete parasite clearance and consequent improved iron absorption and use in the delayed group.

The results of this study will establish a physiologically-based framework for the optimal timing of antimalarial treatment and iron therapy upon which future interventions aimed at improving iron status in malaria-endemic regions can be built, thus helping to reduce the morbidity and mortality and ensure the full neurobehavioral development of the millions of severely anemic children suffering from iron-deficiency and malaria.

**Protocol Overview**

| Title | Acute vs. delayed iron: effect on red cell iron incorporation in severe malaria |
| --- | --- |
| Description | Prospective study of the effect of immediate vs. 4-week delayed oral iron therapy on red blood cell iron incorporation and long-term hematological recovery in Ugandan children aged 6-59 months with anemia (Hb 5-9.9 g/dL) and signs of clinical malaria (positive malaria blood smear + fever in past 24 hours or tem) who present to the Paediatric Assessment Center or Paediatric Acute Care Unit at Mulago Hospital, Kampala, Uganda. Red blood cell iron incorporation on Day 0 and on Day 28 will be measured directly, using iron stable isotopes. |
| Study aims | Aim 1: Identify the sequencing of antimalarial treatment and iron therapy that results in the greatest red cell iron incorporation at the time of initial supplement administration (Day 0 incorporation immediate group vs. Day 28 incorporation delayed group).  Aim 2: Determine whether long-term hematological recovery is impacted by immediate vs. delayed iron (Change in hemoglobin and other iron markers between Day 0 and Day 56). |
| Clinical sites | Mulago Hospital, Kampala, Uganda. |
| Laboratory sites | **Infectious Diseases Institute Core Lab (Mulago Hospital):** Routine onsite testing, including assessment of complete blood counts and preparation of thick and thin smears for malaria parasites. Core lab personnel will also centrifuge blood samples and aliquot plasma to be sent to the U. S. for analysis of iron and inflammatory indicators and will prepare red blood cell pellets for shipment to the U.S. for isotopic analysis.  **Dr. John’s research laboratory in McGuire Translational Research Facility at the University of Minnesota:** Assessment of iron indicators [ferritin, transferrin receptor (TfR), hepcidin] and inflammatory markers C-reactive protein (CRP) and alpha-1-glycoprotein (AGP) in plasma with immunoassay.  **Laboratory of Dr. Steven Abrams, Baylor College of Medicine:** calculation of isotope enrichment fractions in red blood cells using inductively coupled mass spectrometry (ICP-MS). |
| Study participants | Consecutive sample of 100 anemic (Hb 5.0 – 9.9 g/dL) Ugandan children aged 6-59 mo with clinical malaria (positive blood smear and fever in past 24 hours). |
| Selection criteria | Inclusion criteria:   1. 6 - 59 months of age 2. Hemoglobin 5.0 - 9.9 g/dL according to HemoCue 3. Fever in past 24 hours or temperature ≥37.5 4. *P. falciparum* on blood smear at Acute Care Unit 5. Residence <50 km of study hospital   Exclusion criteria:   1. Impaired consciousness on physical exam 2. Seizure activity prior to or during admission 3. Acute malnutrition 4. Known sickle cell disease |
| Study design and procedure | **Day 0**: A venous blood sample is routinely taken from children who present in Acute Care with clinical malaria and anemia (hemoglobin <10 g/dL). Once informed consent is obtained from the caregiver, drops of whole blood will be used to assess erythrocyte protoporphyrin (EP). The EP result will be known immediately. If the child is iron-deficient (EP ≥ 80 µmol/molheme), he or she will be randomized to receive iron before discharge from the hospital (immediate group) or 4 weeks later (delayed group). Iron and inflammatory markers (ferritin, sTfR, hepcidin, reticulocytes, CRP, AGP) will be assessed in each child’s baseline blood sample.  Once consent is obtained and the child is enrolled in the study, each child will be given an oral dose of stable, non-radioactive iron isotope 57Fe mixed with fruit juice,(mango or orange) following a two-hour fast. Caregivers of children in the immediate group will be instructed how to properly administer the first dose of oral iron therapy to be given the following day (Day 1). Iron therapy will consist of a 4-week course of daily ferrous sulfate syrup, based on 2 mg iron/kg body weight.  **Day 28 follow-up visit**: All study children will return to the hospital. A finger prick blood sample will be taken for hemoglobin assessment and for preparation of a Giemsa smear. A venous blood sample will be drawn from all children and sent to Core Lab. Plasma will be collected for assessment of iron and inflammatory markers, and red blood cells will be collected and sent for isotopic analysis. After a two-hour supervised fast, all children will then receive an oral dose of stable, non-radioactive iron isotope 58Fe in an identical manner as on Day 0. Caregivers of children in the delayed iron group will be instructed how to administer the first daily dose of iron syrup to be given the following day (Day 29).  **Day 56 follow-up visit**: On Day 56 all children will return to the hospital for a final finger-prick blood sample (Hb and Giemsa) and venous blood draw (plasma markers plus isotopic analysis) and assessment.  Home monitors will conduct bi-weekly home visits of each child during the 56-day study period to ensure supplement compliance and to assess and record any side effects. Any ill child will be referred to Mulago Hospital and will be provided transportation free of charge. |
| Outcomes | Aim 1: Primary outcome: Percentage of red cell iron incorporation at the time of initial iron supplement administration (Day 0 incorporation immediate group vs. Day 28 incorporation delayed group)  Secondary outcomes: Association between change in plasma iron and inflammatory indicators (EP, ferritin, sTFR, hepcidin, reticulocytes, CRP, AGP; all continuous) and change in percentage red cell iron incorporation in each treatment group between Day 0 and Day 28.  Aim 2: Primary outcomes: Change in hemoglobin from Day 0 to Day 56 in immediate vs. delayed groups.  **Secondary outcomes:** Change in iron markers (EP, ferritin, sTFR, hepcidin) from Day 0 to Day 56 in immediate vs. delayed groups. |

**Significance**

Approximately 25% of the world’s population is anemic.^1^ Globally, iron deficiency and malaria are primary contributors to this anemia, and where the conditions coexist, the resulting anemia is often severe and disproportionately affects young children. The rapid growth of children < 36 months, combined with diets low in bioavailable iron and high in compounds that inhibit iron absorption, make these children vulnerable to dietary iron deficiency, while naïve immune systems with little acquired immunity permit malaria to have particularly harmful health effects. Administering iron in malaria-endemic areas, however, may be dangerous, as excess iron can enhance the growth and pathogenicity of microorganisms.^2^ The established neurobehavioral consequences of untreated iron deficiency, however, necessitate development of effective and safe strategies to provide iron in areas where malaria and other infections are prevalent. Recent research, italicized below, suggests that identification of the sequencing of iron and antimalarial therapy that optimizes iron status and reduces morbidity risk may lay the foundation of any successful strategy

- The *current recommendation for combined treatments for malaria and iron deficiency is not working.* Despite the World Health Organization’s current recommendation that infants and children who have malaria and are diagnosed with iron deficiency or severe anemia “be treated with antimalarial and, where appropriate, antibiotic therapy as well as iron therapy,”^3^ evidence of the effect of combined iron and antimalarial treatment on hemoglobin concentration in young children in malaria-endemic areas has been mixed, and reports of increased malaria parasitemia and clinical episodes are frequent.^4-6^
- *Paradigm shift: Clearing malaria and associated inflammation first and then providing iron may enhance the efficacy and reduce the risk of both interventions*. Strong evidence of hepcidin concentrations^7^ and red blood cell iron incorporation in *P.-falciparum*-infected children^8^ supports the hypothesis that both antimalarial treatment and iron therapy would be most efficacious if not administered concurrently and that a delay of approximately four weeks after antimalarial treatment would ensure reduction of inflammation and optimal incorporation of oral iron.
- If our hypotheses that delaying iron until 4 weeks after antimalarial treatment optimizes red blood cell iron incorporation and improves long-term hematological recovery proves to be correct, malaria and iron deficiency intervention programs adopting this sequencing strategy could both reduce the immediate morbidity and mortality as well as ensure the full neurobehavioral development of the millions of iron-deficient children who live in malaria-endemic regions.

**INNOVATION**

- By employing direct methods to track oral iron and its incorporation into red blood cells in children receiving iron concurrently with antimalarial therapy or four weeks later, the proposed study will establish the sequencing of these interventions that optimizes their efficacy, providing a physiologically-based framework for future malaria and iron deficiency treatment programs.
- This 4-week delay has not been tested directly using both stable iron isotopes and a randomized design, nor has it been investigated in children with severe anemia and clinical malaria, in whom the mortality burden is great.

**Background**

As in many regions of the world, including sub-Saharan Africa, Southeast Asia, and Central and South America, where iron deficiency and malaria coexist, the prevalence of anemia among children younger than three years living in Uganda is an estimated 66-77%.^9^ The results of a recent large, randomized, placebo-controlled trial on Pemba Island, Tanzania^10^ underscored the potential danger of providing iron in malaria-endemic regions to address this prevalent anemia, finding that children who received iron supplements were more likely to experience a serious adverse event, i.e., death or hospitalization, than children who received placebo. Although a sub-study of the trial indicated that the harmful effect of iron was limited to children who were iron-replete at baseline, the question of how to treat both conditions safely and efficaciously in malaria-endemic areas remains largely unanswered. Although several studies report modest (~ 1g/dL) gains in hemoglobin concentration among children receiving concurrent iron and antimalarial therapy in accordance with WHO guidelines,^4-6,11^ this gain is typically not sufficient to cure the profound anemia of the majority of children. Further, many authors report non-statistically significant increases in malaria indices among children receiving iron, including increased parasitemia^4, 5^and increased risk of malaria attack.^6^Noting that a larger proportion of the iron-treated patients failed to clear their parasitemia, Nwanyanwu et al. concluded that antimalarial treatment should be given first, followed by iron therapy, if needed.^5^

The pathophysiology of anemia resulting from co-existing iron deficiency and malaria infection supports this conclusion.The anemia of malaria is complex, with hemolysis of parasitized red blood cells, clearance of both parasitized and non-parasitized red blood cells by reticulo-endothelial macrophages, and impaired bone marrow activity all contributing.^12,13^ Iron that would normally be recycled from degraded red blood cells and transported to the bone marrow for erythropoiesis is instead sequestered in reticulo-endothelial stores under the influence pro-inflammatory cytokines IL-1, IL-6, TNF-α, IFN-γ, the same cytokines implicated in contributing to the alterations in iron metabolism characteristic of the anemia of chronic disease.^14-17^ The antimicrobial hepatic protein hepcidin, an integral regulator of iron metabolism that serves as the soluble messenger among the sites of iron absorption, erythropoiesis, and storage,^18^also plays a key role in these inflammatory changes. Hepcidin concentrations rise during iron sufficiency, down-regulating intestinal iron absorption, and fall during iron deficiency, up-regulating iron absorption. Hepcidin is also a positive acute phase response protein whose concentrations are up-regulated during inflammation under the influence of IL-6^19^--a finding that is thought to explain the alterations of iron metabolism (hypoferremia, hyperferritinemia, anemia) during infectious and non-infectious inflammatory states. Hepcidin acts primarily by binding and degrading the iron-exporting protein ferroportin,^20,21^ blocking iron efflux from intestinal and reticulo-endothelial cells. Multiple studies have confirmed that hepcidin concentrations increase with *P. falciparum* infection^7, 22,23^ and that even mild increases in serum hepcidin can lead to profound disturbances in iron metabolism, including marked hypoferremia, increased serum ferritin, and reduced incorporation of hemoglobin into reticulocytes.^7^

These changes appear to be resolved approximately four weeks after successful antimalarial treatment. One recent study of *P. falciparum*-infected, hospitalized Tanzanian children aged 2 mo-13 y who were anemic and febrile found that urinary concentrations of hepcidin at baseline were high and were associated with hypoferremia and increased serum ferritin.^7^ Antimalarial treatment rapidly decreased hepcidin concentrations and reversed hypoferremia. Serum iron concentrations and transferrin saturation rose rapidly, but significant changes in total iron binding capacity and serum ferritin were observed only after four weeks. Concentrations of CRP and pro-inflammatory cytokines IL-1Ra and IL-6 paralleled the changes in hepcidin, falling significantly two days after antimalarial treatment and declining even further by Week 4. The authors concluded that marrow suppression in patients with febrile malaria is rapidly reversible with efficacious antimalarial treatment and that iron therapy might only be effective once hepcidin concentrations decline. A study of red cell iron incorporation in young Gambian children treated for uncomplicated malaria and given a 30-day course of iron similarly demonstrated that iron therapy might be most efficacious if given 2-4 weeks after antimalarial treatment.^8^Using iron stable isotopes, researchers found that children recovering from malarial anemia exhibited significantly lower red cell iron incorporation at baseline and at Day 15 (baseline: 8% vs. 28%, p<0.001; Day 1: 14% vs. 26%, p=0.04), but had a greater hemoglobin concentration on Day 15 and Day 30 than children with iron deficiency anemia alone, leading researchers to conclude that children in the post-malaria group met their immediate iron needs for erythropoiesis with recycled iron liberated from reticulo-endothelial stores after antimalarial treatment rather than from the oral supplement. Whether this hemoglobin recovery would have been even greater if the iron therapy had been delayed until red cell iron incorporation was more optimal, i.e., some time after Day 15, is unknown. Also unknown is whether iron absorption and subsequent incorporation into red cells would follow similar patterns in children with severe anemia and clinical malaria in whom inflammation-induced iron trapping and subsequent hypoferremia is likely more profound. To date, red cell iron incorporation during malaria infection has only been investigated in two populations, Gambian children with uncomplicated malaria, as described, and young Beninese women of childbearing-age with subclinical malaria.^24^

**Study Procedure**

**Overview:** We will recruit Ugandan children aged 6-59 months with anemia (hemoglobin 5 – 9.9 g/dL) and with clinical evidence of malaria who present to the Paediatric Assessment Center and the Paediatric Acute Care Unit at Mulago Hospital in malaria-endemic Kampala, Uganda. All children will be treated for malaria, and any child found to be iron-deficient [erythrocyte protoporphyrin (EP) ≥ 80μmol/mol heme] will be randomized to start a 4-week course of daily iron therapy (2 mg/kg) immediately on Day 0 (immediate group) or 4 weeks later on Day 28 (delayed group). All children will return to the hospital on Day 56 for final assessment (**Figure 1**). To determine the percentage of oral iron incorporated into red blood cells on Day 0 and Day 28, all children will be given an oral dose of iron stable isotope, 57Fe, on Day 0 and an oral dose of a different iron stable isotope, 58Fe, on Day 28. Calculation of isotopic enrichment of the red blood cells 28 days after dosing will permit comparison of red blood cell iron incorporation at the time of initial administration of the iron supplement between the treatment groups (Day 0 incorporation for immediate group vs. Day 28 incorporation for delayed group, Aim 1). Comparison of the change in hemoglobin and other iron markers (erythrocyte protoporphyrin, ferritin, soluble transferrin receptor, hepcidin, reticulocytes) from Day 0 to Day 56 between treatment groups will enable assessment of how immediate vs. delayed iron impacts long-term hematological recovery and iron status (Aim 2).

Study Recruitment and Enrollment: We will recruit a consecutive sample of 100 children with Hb 5.0-9.9 g/dL and between the ages of 6-59 months who present to the Pediatric Acute Care unit at Mulago Hospital, Kampala, Uganda. The inclusion and exclusion criteria are as follows:

Inclusion criteria:

1.) 6 - 59 months of age

2.) Hemoglobin 5.0 - 9.9 g/dL according to HemoCue

3.) Fever in past 24 hours or temperature > 37.5

1. *P. falciparum* on blood smear at Acute Care Unit
2. Residence < 50 km of study hospital

Exclusion criteria:

1. Impaired consciousness on physical exam
2. Seizure activity prior to or during admission
3. Acute malnutrition
4. Known sickle cell disease

**Day 0 Procedure:** Children younger than 5 years who present to the Paediatric Assessment Center or Paediatric Acute Care Unit with a positive blood smear will have a finger-prick blood sample for hemoglobin and preparation of Giemsa smear as part of routine care. If hemoglobin is 5.0 – 9.9 g/dL, the Giemsa smear is positive, and the child lives < 50 km from the hospital, the child will be eligible for the study. After informed consent for our research study is obtained (see Informed Consent, below), a venous blood sample will be drawn. Blood samples are routinely drawn on children with this clinical presentation as part of standard of care. After informed consent for our research study is obtained (see Informed Consent, below), drops of whole blood will be dispensed for immediate determination of EP by hematofluorometer (Aviv Biomedical, Lakewood, NJ) and for preparation of a reticulocyte smear. The remaining blood will be sent to Corelab for collection of plasma. Children found to be iron-deficient (EP ≥ 80 µmol/molheme) will be randomized to start 4 weeks of daily iron therapy at baseline, once oral medication can be tolerated (immediate group), or 4 weeks later on Day 28 (delayed group). Any child who is not iron-deficient will not receive iron therapy. Upon enrollment all children will be assigned a randomly generated, unique study identification number that will link clinical forms and samples. Caregivers will be given a clinical follow-up schedule, with dates of bi-weekly home visits and Day 28 and Day 56 hospital visits indicated. On Day 0, participants will be given 2500 USh for transport home. On both the Day 28 and Day 56 visits, participants will receive reimbursement commensurate in value to 15,000 USh. 5000 USh will be given to cover transportation costs to and from the hospital and any cell phone air time needed in the preceding 4 weeks to report illness, and a food package containing oil, rice, and/or sugar valuing 10,000 USh.

**Informed consent:** Once each eligible child is medically stable, a study representative will conduct the informed consent process. The iron study consent form describing the study, including risks and benefits of study participation, will be presented in either Luganda or English, depending on the parent’s preference. Translators will be available if needed. Study staff will explain that not participating in the study or deciding to stop participation will not jeopardize the child’s care. Copies of signed consent forms will be kept on file at the study research offices at Mulago Hospital.

**Randomization:** A list of 100 random, 3-digit numbers will be computer-generated at the University of Minnesota. These numbers will serve as unique study identification numbers. Treatment group (either immediate or delayed) will be then be randomly assigned to each number, in blocks of four. The treatment group for a particular study ID will be placed in a sealed envelope. Envelopes will be opened sequentially by study personnel at patient enrollment.

**Clinical treatment:** Clinical treatment of all children who are enrolled in the study will be overseen by Dr. Ezekiel Mupere. Ugandan national guidelines will be followed for treatment of malaria, presently oral quinine for hospitalized patients and artemethar combination drugs for outpatients. Initial labs and testing will be done by study physicians and nurses, but clinical care of all patients will be given by the house staff and attending physicians of Mulago Hospital. Other co-infections and medical problems will be treated according to standard protocols of Mulago Hospital. Thick and thin blood smears will be prepared for all enrolled children to look for malaria parasites and density of parasitemia, calculated using parasites per 200 WBC.

**Figure 1: Study design I**

**Isotope administration:** Iron stable isotope 57Fe (Trace Sciences International) will be converted into an aqueous solution of ferrous sulfate, as described by Ames et al.^25^ Following baseline blood draw and a supervised, 2-hour fast, all enrolled children will receive an oral dose of 57Fe (4 mg)^8^ along with a crushed tablet of vitamin C (50 mg) in 30 mL of fruit juice. Children will be observed for 2 hours after administration of the isotope, and any spilling or vomiting will be recorded. On Day 28, after venous blood draw, an oral dose of 58Fe (1.5 mg) will be given using the identical procedure as for 57Fe. Each 1.5 mg dose of 58Fe will be spiked with 2.5 mg liquid ferrous sulfate to make the total iron content of each dose of 57Fe and 58Fe equivalent. Isotopic enrichment of the blood samples will be calculated by consultant Dr. Steven Abrams, Baylor College of Medicine, as previously described by his research group.^26^These calculations will provide a direct estimate of the proportion of iron isotope incorporated into red blood cells on Day 0 and Day 28. For the purpose of these calculations, each child’s blood volume will be estimated at 80 mL/kg.^26^

**Home visits:** All enrolled children will receive bi-weekly home visits to ensure compliance and to inquire about the overall health of each child. Ill children will be referred to the hospital for care, with transportation provided. Any iron side effects, e.g., constipation, vomiting, diarrhea, will be recorded.

**Sample collection:** *Phlebotomy:* A venous blood sample of no more than 5 mL is routinely drawn from patients with this clinical presentation as part of standard of care. Whole blood will be used for assessment of EP and reticulocytes. This Day 0 blood, along with blood samples drawn at the Day 28 and Day 56 hospital visits will be centrifuged, and plasma collected at the CoreLab located onsite at Mulago Hospital. Plasma will be stored at -80^o^C and then shipped on liquid nitrogen to the University of Minnesota for quantification of iron and inflammatory markers (ferritin, sTfR, hepcidin, CRP, AGP) by immunoassay in the lab of Dr. Chandy John. A finger-prick blood sample will also be drawn on Days 28 and Day 56 for hemoglobin assessment by HemoCue and for Giemsa stain preparation. Red blood cell pellets collected at Day 28 and Day 56 will be re-suspended in saline, stored at -80^o^C, and shipped to Baylor College of Medicine for isotopic analysis by inductively coupled plasma mass spectrometry (ICP-MS) in the laboratory of Dr. Steven Abrams.

*Stool sample:* Per Mulago Hospital guidelines, a stool sample will be collected from each child on Day 0 and analyzed for helminths. Any child found to be infected will be treated with mebendazole.

**Statistical analysis:** For our primary outcome for Aim 1,we will compare Day 0 red cell iron incorporation in the immediate group with Day 28 red cell iron incorporation in the delayed group using a two-sample t-test. Secondary analyses will adjust for baseline characteristics of the children, including age, sex, height, and weight. For our secondary outcome of the association of the change in plasma iron and inflammatory indicators with the change in percentage red cell iron incorporation in each treatment group, strength of trends will be assessed by Spearman correlations. We will also compare the mean change in red cell iron incorporation from Day 0 to Day 28 in the immediate vs. delayed groups using separate multivariate regression models for each indicator. In each model, the change in red cell incorporation will be the outcome variable, and treatment group, Day 0 hemoglobin, and the mean changes from Day 0 to Day 28 in ferritin, sTfR, EP, hepcidin, AGP, or CRP as predictor variables. Models will be further adjusted for sex, age, and anthropometric indices.

**Sample size calculations:** Based on variation between children in 57Fe incorporation reported in Doherty et al.,^8^40 children are needed in each group to provide a clinically significant, detectable difference in red cell incorporation between groups of 4.5%, assuming 80% power and alpha=0.05 (**Table 1**). To account for 20% loss to follow-up, we will recruit 50 children in each group.

**Table 1: Power and sample size for Aim 1**

|  | Detectable difference | |
| --- | --- | --- |
| n per group | Power 80% | Power 90% |
| 30 | 5.2 | 6.0 |
| 33 | 5.0 | 5.8 |
| 40 | 4.5 | 5.2 |
| 43 | 4.3 | 5.0 |
| 50 | 4.0 | 4.6 |
|  |  |  |

For our primary outcome for Aim 2 of the change in hemoglobin from Day 0 to Day 56 in the immediate vs. delayed iron groups, we will compare mean 56-day change (Day 56 minus Day 0) in hemoglobin between the treatment groups using a two-sample t-test. Secondary analyses will adjust for baseline characteristics of children, including age, sex, anthropometric indices, and stool helminth count. For our secondary outcome of changes in iron indicators between Day 0 and Day 58, we will compare the mean 56-day change in each iron indicator (EP, ferritin, sTfR, hepcidin) between treatment groups using a two-sample t-test for each indicator. Secondary analyses will adjust for age, sex, anthropometric indices and helminth count.

**Sample size calculations:** Assuming a SD in the mean change in hemoglobin to be 1.5 g/dL and alpha = 0.05, with 50 children in each treatment group, we would have 90% power to detect a difference of 1.10 g/dL in the mean 56-day change in hemoglobin concentration between groups, assuming 20% loss to follow-up.

**Protection Of Human Subjects**

**Proposed involvement of human subjects:** This study proposes to use iron stable isotopes to study the effect of immediate vs. 4-week delayed iron therapy to treat iron-deficiency in 100 moderately to severely anemic Ugandan children with malaria who present at the Pediatric Acute Care Unit at Mulago Hospital in Kampala, Uganda. All children will be treated for malaria according to national guidelines and then randomized to receive a 4-week course of iron therapy that starts concurrently with antimalarial treatment (immediate group) or 4 weeks after antimalarial treatment on Day 28 (delayed group). Children in both groups will receive 57Fe at baseline and 58Fe at 28 days follow-up. Proposed blood draws will be at baseline (as part of routine hospital admission), Day 28, and Day 56. This design will permit comparison of percent of iron incorporated into red cells at the time of initial supplement administration in the immediate vs. delayed groups (Aim 1) and also long-term hematological recovery between the two treatment groups (Aim 2).

**Characteristics of the subject population:** Moderate to severe anemia resulting from iron deficiency and malaria infection disproportionately afflicts very young children in sub-Saharan Africa and is a primary cause of hospitalization and mortality. Neither iron deficiency nor malaria is common in very young infants until 6 months of age, when conferred immunity from malaria via maternal antibodies wanes and birth iron stores are depleted. The rapid growth of children between 6-59 months, coupled with diets deficient in absorbable iron, places them at high risk of iron deficiency. This period of growth corresponds with peak vulnerability to malaria infection before immunity to the parasite is achieved. We proposed to enroll 100 participants, as this number provides sufficient statistical power to observe a clinically meaningful difference in red cell iron incorporation in the immediate vs. delayed groups.

**Sampling plan:** We will recruit a consecutive sample of children 6-59 months with anemia (hemoglobin 5-9.9 g/dL) and malaria. This is a group of children that commonly presents to the Pediatric Acute Care Unit at Mulago Hospital. All participants will receive extensive follow-up, including bi-weekly home visits and transportation to the hospital for scheduled visits and for any intervening illness. Within this group of moderately to severely anemic children, no distinction will made based on gender or minority. We will exclude children who live far enough from the hospital that complete follow-up would be difficult; children with hemoglobin < 5 g/dL, as these children receive transfusion upon admission; children who are not anemic (hemoglobin ≥ 10 g/dL), as our project focuses on iron absorption and utilization in anemia; and children with signs of cerebral malaria, including impaired consciousness or recent seizure activity.

**Involvement of special classes of subject:** We will be enrolling anemic Ugandan children aged 6-59 months, as the public health problem of coexisting iron deficiency and malaria infection is greatest at this age and in this part of the world.

**Study group assignment:** All children who are found to be iron-deficient (elevated erythrocyte protoporphyrin) will be randomized to receive iron concurrently with antimalarial treatment, which is the current standard of care, or 4-weeks after anti-malarial treatment. We hypothesize that children receiving delayed iron will exhibit greater long-term hematologic recovery and iron status due to more complete parasite clearance and resolution of inflammation at the time of initial supplement administrations, despite the delayed administration of iron.

All children will receive a single dose of iron stable isotope 57Fe (4 mg) at baseline and 58Fe (1.5 mg) at Day 28. Stable isotopes are a safe and accessible way to measure mineral metabolism. They are used regularly to investigate iron absorption in many vulnerable groups, including premature infants and pregnant women. The 4-week course of iron given to all iron-deficient children will be in the form of liquid ferrous sulphate, at a dose of 2 mg iron per kg body weight, as recommended by the World Health Organization.

**Collaborating sites:** This study will take place at Mulago Hospital in Kampala, Uganda. Mulago is the largest referral hospital in Uganda, and our group has collaborated with pediatric malaria researchers there for more than a decade. For the current study, on-site collaborators will be responsible for patient enrollment and care while in the hospital, administration of study interventions, obtaining and proper care of study samples, and coordination of follow-up for the duration of the study period. Our on-site data team will ensure complete data entry from clinical forms, collected in the hospital and in the field, and will conduct periodic quality checks on data integrity and completeness. All study forms will be kept in a locked desk in the Cerebral Malaria Team office, and the study database will be password-protected.

**Description of research material obtained**: Blood samples will be collected from all enrolled children at baseline, at Day 28, and at Day 56. From these blood samples, plasma will be collected, frozen, and shipped to the US for analysis of iron markers. The red blood cell pellet from each blood sample will be suspended in saline, frozen, and also shipped to the US for analysis of isotope ratios. Data from clinical forms will be kept in a password-protected database, which is routinely backed up. Paper copies of forms will be kept in a locked office. Any shared data set will be stripped of all patient identifiers.

**Description of data:** We expect to collect data on the concentrations of iron and inflammatory markers in the plasma of all enrolled children at 3 time points. We also expect to gain values of red cell iron incorporation at Day 0 and Day 28. We will further have morbidity data for the 56-day duration of the study, including the number of malaria episodes and fevers.

**Linkages and access to subject identities:** The study coordinator, home visitors, nurses, and data manager will have access to identifiable information, as it is necessary to ensure full follow-up, patient care, and accurate data entry and storage.

**Collection of specimens, records, or data:** Each participant will be given a unique study id upon enrollment. These id’s will be randomly generated, as described in the Randomization section. Upon collection, all specimens, records, and forms for each participant will be marked and linked with this unique study id. Only the study coordinator, home visitors, nurses, and data manager will be able to link study id with identifiable information, as this linkage is critical for optimal patient care while at the hospital and for complete home follow-up.

**Potential risks to subjects:**

*Potential risk: Iron therapy*

The primary risk of iron therapy in a malaria-endemic area is the risk of increased episodes of malaria. We will be giving iron only to those children who are iron-deficient. A recent, large, randomized controlled trial in Pemba, Tanzania, showed an increased risk of serious adverse events (hospitalizations and mortality) in children given iron/folic acid. A sub-study revealed, however, that this risk was limited to those who were iron-sufficient and received iron, while children who were iron-deficient (as defined by an elevated erythrocyte protoporphyrin) had significantly *fewer* serious adverse events. We do not expect to see an elevated malaria risk with the WHO-recommended dose that we are giving for several reasons: 1) We are only giving iron the iron-deficient children. These children benefitted from iron in the Pemba study; 2) We will be providing bi-weekly home visits and access to health care, which was not available on the main Pemba study; 3) We will instruct caregivers to provide the iron with a small amount of food. No instruction with regard to timing of food intake was given in the Pemba study, and it is possible that concurrent consumption of food would slow the “bolus” absorption imposed by a large dose of supplemental iron given by itself. Such a bolus dose has been hypothesized to increase the amount of free, or non-transferrin-bound iron which may exacerbate existing malaria; and 4) Unlike the Pemba study, we will not be giving folic acid, which can reduce the efficacy of certain anti-folateantimalarials, including sulphadoxine-pyramethamine, the antimalarial used in the Pemba study. For all of these reasons, we believe the risk of serious adverse events due to iron therapy is low in our population.

The potential risks described above pertain primarily to the daily, liquid ferrous sulphate syrup that each iron-deficient child will receive for 4 weeks. The amount of iron given in the stable isotope form is minute compared to the daily supplemental amount and unlikely to cause any adverse effects. Stable isotopes are naturally occurring forms of a mineral, in this case iron, which can be used as a tracer in the body. There are no known risks to the stable isotopes.

In any setting, iron may be associated with gastrointestinal distress. We will monitor this side-effect through parental reports in bi-weekly home visits and discontinue therapy if discomfort persists. All potential side-effects will be monitored by the study’s DSMB (see DSMB below).

*Potential risk: Lab test collection:* The study protocol calls for three venous blood draws—one at baseline, one at Day 28, and one at Day 56. The baseline blood draw is part of the routine care for any admitted patient with severe anemia and malaria. The only risk to study participants are those of venipuncture, including persistent bleeding, pain, or infection. Experienced nurses and medical officers will ensure that all testing is done in a sterile manner. We have documented no significant side effects from the venipuncture we have done previously at Mulago Hospital, so we do not consider this testing to be of significant risk to the study subjects.

**ADEQUACY OF PROTECTION AGAINST RISKS**

**Plans for recruitment and informed consent:** Study participants will be recruited in the Pediatric Acute Care Unit of Mulago Hospital. Informed consent will be obtained from parents or guardians of all study participants, with information on risks and benefits being provided and consent forms being given to the parents or guardians in the local language. Data will be stored in a locked office accessible only to study personnel. Caregivers of potential participants will be informed that their child can leave the study at any time without jeopardizing the care they will receive at Mulago Hospital. No study-specific tests will be administered before informed consent is obtained.

**Circumstances of consent:**Consent will be sought and obtained from caregivers bringing eligible children into the Pediatric Acute Care Unit. Once the patient is medically stabile, the Medical Officer on duty will approach the caregiver and inform him or her about the study and will provide a full description of risks and benefits of study participation. The consent form will be available in both English and Luganda, the local language. The caregiver will acknowledge consent by signing the form.

**Plans for protection against risk:**

*Iron therapy:* Bi-weekly home visits will be made for every study participant. At these home visits, any ill child, including those with clinical signs of malaria, will be referred and given transportation to Mulago Hospital for care. Any child with smear-confirmed malaria infection will receive treatment. Transportation and treatment are provided free of charge. At biweekly home visits, study monitors will also verify that the iron is taken appropriately, will record any outside clinic visits, and will monitor any adverse events. A Data Safety and Monitoring Board (See DSMB, below) will be established to ensure human subjects’ protection throughout the study period.

*Blood draws:* Experienced nurses and medical officers will ensure that all testing is done in a sterile manner. We have had no adverse events from blood draws in any previous or ongoing studies at Mulago.

**Plans for medical or professional intervention:** Any enrolled child found to be ill during the study period will receive both transportation to the hospital and medical care free of charge. To ensure the safety of trial participants, study investigators are monitoring, recording, and reporting both adverse events and serious adverse events. An adverse event (AE) will be defined as any untoward medical response to the iron therapy itself, including, but not limited to, constipation, abdominal pain, and diarrhea. These symptoms will be recorded by home monitors. Home visit forms will be returned to the study office and reviewed by study medical officers on a daily basis. A *serious adverse event* is any untoward medical occurrence that happens after initial hospitalization and discharge that results in death, is life-threatening, requires hospitalization, or involves a substantial overdose of iron (consumption of more than 10 times the prescribed dose at a single time*)*.

In the event of a serious adverse event, the study medical officer will be notified immediately and will complete a Serious Adverse Event Reporting form. A copy of this form will be placed in the child’s folder and another copy will be placed in a SAE folder, to be kept in the office. The Medical Officer will also inform the site PI of the SAE within 48 hours. The site PI will then inform the U.S. PI, ideally within 48 hours, but in no more than 7 calendar days. The U.S. PI will relay SAE information to the DSMB chair in no more than 15 calendar days from the time of the event. The PIs will also submit periodic reports of all SAE’s (quarterly for Makerere University School of Medicine Research Ethics Committee, yearly for University of Minnesota Institutional Review Board.)

If a subject is withdrawn because of a serious adverse event that does not result in death, the subject will be followed and treated by the medical officer until the abnormal parameter or symptom has resolved or stabilized.

**Potential benefits of the research:**With the proposed study, children with elevated erythrocyte protoporphyrin will receive a 4-week course of ferrous sulphate. Improvement in iron status has been associated with improved neurobehavioral development in numerous studies, and giving iron to children with elevated erythrocyte protoporphyrin living in malaria-endemic areas has been shown to be protective against hospitalizations and mortality. Enrolled children will have no direct benefit from the hematologic and isotopic studies done, but will have minimal risk from these studies. Free health care and transportation to Mulago hospital will be provided to all enrolled children for any illness in the course of the study.

**Relationship of risks to anticipated benefits:** The risk of iron therapy in malaria-endemic areas is likely confined to those children who are not iron-deficient, as described above. We do not believe that the WHO-endorsed dose of iron that we are providing to iron-deficient children will impose a risk. Rather, we believe that the iron will be protective against serious adverse events and perhaps will help to ensure improved neurobehavioral development. The risk of complications from venipuncture is also small in comparison to the established benefit of providing iron to iron-deficient children.

**Importance of the knowledge gained:** The present study will provide important and direct physiologic evidence of the best sequencing of iron and anti-malarial treatment for iron-deficient children living in malaria-endemic areas. Delaying iron until after resolution of inflammation and parasite clearance may result in better absorption, thus increasing the efficacy of iron therapy. This information will help guide malaria and iron intervention programs and potentially help to improve neurobehavioral development of millions of children, as iron deficiency and malaria coexist in many regions of the world, including sub-Saharan Africa, Southeast Asia, and Central and South America.

**Relationship of risks to the knowledge gained:** Iron deficiency is an established cause of cognitive impairment. Establishing direct, physiological evidence for the best sequencing of interventions has the promise to improve millions of lives and may in fact lessen any risk of giving iron in malaria-endemic areas.

**Data and safety monitoring plan:** A Data Safety Monitoring Board will be established to provide external, objective advice regarding the safety and efficacy of the administered interventions. The Board will be comprised of experts in clinical trials, biostatistics, iron, and child health, as well as one lay person. At least one Board member will be Ugandan. Board members will be independent from the study and will meet in open and closed sessions at the start of the study, when half of the expected number of children have completed the study, and at the completion of the study to evaluate study data and provide recommendations pertaining to participant safety and trial conduct to investigators.

While the DSMB will meet at these appointed times to review SAE’s, to advise on issues of protocol adherence, to review recruitment and follow-up rates, and to advise the investigators on pertinent safety issues, formal stopping rules are not in place for this study on account of the small sample size and resulting limited power for an interim analysis to make meaningful comparisons between treatment groups. We have the same treatment groups (immediate and delayed iron) in more severely ill children (cerebral malaria or severe malarial anemia) in our ongoing, larger (n=300) U01 study at Mulago Hospital and have not observed SAE differences that exceed stopping rule thresholds in that study to date.

**ClinicalTrials.gov requirements:** The proposed study has been registered with ClinicalTrials.gov by the PI, as required. Updates regarding recruitment status or protocol changes will be made in a timely manner.

**References cited**

1. World Health Organization. Vitamin and Mineral Nutrition Information System website. Accessed October 6, 2011 at: <http://www.who.int/vmnis/database/anaemia/anaemia_data_status_t2/en/index.html>
2. Prentice AM. Iron metabolism, malaria, and other infections: what is all the fuss about? J Nutr. 2008;138(12):2537-2541.
3. Conclusions and recommendations of the WHO Consultation on prevention and control of iron deficiency in infants and young children in malaria-endemic areas. Food Nutr Bull. 2007;28(4 Suppl);S621-7.
4. Desai MR, Mei JV, Kariuki SK et al. Randomized, controlled trial of daily iron supplementation and intermittent sulfadoxine-pyramethamine for the treatment of mild childhood anemia in western Kenya. J Infect Dis. 2003;187(4):658-66.
5. Nwanyanwu OC, Ziba C, Kazembe PN, Gamadzi G, Gandwe J, Redd SC. The effect of oral iron therapy during treatment for *Plasmodium falciparum* malaria with sulphadoxine-pyramethamine on Malawian children under 5 years of age. *Ann Trop Med Parasitol*. Dec 1996;90(6):589-595.
6. Verhoef H, West CE, Nzyuko SM et al. Intermittent administration of iron and sulfadoxine-pyramethamine to control anaemia in Kenyan children: a randomised controlled trial. Lancet. 2002;360(9337):908-14.
7. de Mast Q, Nadjm B, Reyburn H et al. Assessment of urinary concentrations of hepcidin provides novel insight into disturbances in iron homeostasis during malarial infection. J Infect Dis. 2009;199(2):253-62.
8. Doherty CP, Cox SE, Fulford AJ et al. Iron incorporation and post-malaria anaemia. *PLoS ONE*. 2008;3(5):e2133.
9. World Health Organization. Vitamin and Mineral Nutrition Information System website. Accessed October 6, 2011 at: <http://who.int/vmnis/anaemia/data/database/countries/uga_ida.pdf>
10. Sazawal S. Black RE, Ramsan M et al. Effects of routine prophylactic supplementation with iron and folic acid on admission to hospital and mortality in preschool children in a high malaria transmission setting: community-based, randomised, placebo-controlled trial. Lancet. 2006;367(9505):133-43.
11. vanHensbroek MB, Morris-Jones S, Meisner S et al. Iron, but not folic acid, combined with effective antimalarial therapy promotes haematological recovery in African children after acte falciparum malaria. Trans R Soc Trop Med Hyg. 1995;89(6):672-6.
12. Menendez C, Fleming AF, Alonso PL. Malaria-related anaemia. Parasitol Today. 2000;16(11):469-76.
13. Prentice AM, Ghattas H, Doherty C, Cox SE. Iron metabolism and malaria. *Food Nutr Bull*. Dec 2007;28(4 Suppl):S524-539.
14. Kobune M, Kohgo Y, Kato J, Miyazaki E, Niitsu Y. Interleukin-6 enhances hepatic transferrin uptake and ferritin expression in rats. Hepatology. 1994;19(6):1468-75.
15. Means JT, Jr., Kranz SB. Progress in understanding the pathogenesis of the anemia of chronic disease. Blood. 1992;80(7):1639-47.
16. Othoro C, Lal AA, Nahlen B, Koech D, Orago AS, Udhayakumar V. A low interleukin-10 tumor necrosis factor-alpha ratio is associated with malaria anemia in children residing in a holendemicmalria region in western Kenya. J Infect Dis. 1999;179(1):279-82.
17. Rogers JT. Ferritin translation by interleukin-1 and interleukin-6: the role of sequences upstream of the start codons of the heavy and light subunit genes. Blood. 1996;87(6):2525-37.
18. Leong WI, Lonnerdal B. Hepcidin, the recently identified peptide that appears to regulate iron absorption. *J Nutr*. Jan 2004;134(1):1-4.
19. Nemeth E, Valore EV, Territo M, Schiller G, Lichtenstein A, Ganz T. Hepcidin, a putative mediator of anemia of inflammation, is a type II acute-phase protein. Blood. 2003; 101(7):2461-3.
20. Nemeth E, Rivera S, Gabayan V, et al. IL-6 mediates hypoferremia of inflammation by inducing the synthesis of the iron regulatory hormone hepcidin. *J Clin Invest*. May 2004;113(9):1271-1276.
21. Nemeth E, Tuttle MS, Powelson J et al. Hepcidin regulates cellular iron efflux by binding to ferroportin and inducing its internalization. Science. 2004;306(5704):2090-3.
22. deMast Q, van Dongen-Lases EC, Swinkels DW et al. Mild increases in serum hepcidin and interleukin-6 concentrations impair iron incorporation in haemoglobin during an experimental human malaria infection. Br J Haematol. 2009;145(5):657-64.
23. Howard CT, McKapo US, Quakyi IA et al. Relationship of hepcidin with parasitemia and anemia among patients with uncomplicated Plasmodium falciparum malaria in Ghana. Am J Trop Med Hyg. 2007;77(4):623-6.
24. Cercamondi CI, Egli IM, Ahouandjinou E. Afebrile *Plasmodium falciparum*parasitemia decreases absorption of fortification iron but does not affect systemic iron utilization: a double stable-isotope study in young Beninese women. *Am J ClinNutr*. 2010;92:1385-1392.
25. Ames SK, Gorham BM, Abrams SA. Effects of high compared with low calcium intake on calcium absorption and incorporation of iron by red blood cells in small children. Am J ClinNutr. 1999;70:44-8.
26. Abrams S. Using stable isotopes to assess mineral absorption and utilization by children. Am J ClinNutr. 1999;70:955-64.
